# How does habit strength affect reactions to warning labels? evidence from Chinese consumers’ choices of carbonated beverages

**DOI:** 10.1371/journal.pone.0316189

**Published:** 2024-12-26

**Authors:** Yulian Ding, Yue Yang, Yangyang Sun, Kevin Chen, Lianfeng Hu

**Affiliations:** 1 Institute of Western China Economic Research, Southwestern University of Finance and Economics, Chengdu, China; 2 Zhejiang Province Key Think Tank: Institute of Ecological Civilization, Zhejiang A&F University, Hangzhou, China; 3 College of Economics and Management, Zhejiang A&F University, Hangzhou, China; 4 Center for Agricultural and Rural Development, Zhejiang University, Hangzhou, China; 5 Industry Planning Division, Chengdu Municipal Bureau of Economic and Information Technology, Chengdu, China; Bangor University, UNITED KINGDOM OF GREAT BRITAIN AND NORTHERN IRELAND

## Abstract

Effectively regulating the excessive consumption of sugar-sweetened beverages (SSBs) has been an important task for public health authorities around the world. The rapid increase in SSB consumption in China necessitates robust regulations. This study employed a choice experiment to simulate the market scenario in which a text warning label was presented on SSBs. We draw a sample of 1200 participants from five major cities across China to analyze the warning label’s effectiveness in reducing urban consumers’ purchases of SSBs, with a particular emphasis on how an individual’s habit strength affects their reaction to the warning label. Our analysis unveiled varying levels of habit strength among respondents: 35.33% showed weak habits, 56.25% displayed moderate habits, and 8.42% demonstrated strong habits. The random parameters logit model results showed a significant overall decrease in SSB purchases when a warning label was present, indicated by a significant negative coefficient associated with the label. However, when examining the impact of warning labels across different habit strengths, a significant effect was observed only in the moderate habit group. Furthermore, within this group, the warning label was more effective for those with correct health knowledge and who were not overweight or obese.

## 1. Introduction

Global overweight and obesity rates have surged dramatically over the past decades. Increased consumption of energy-dense foods high in sugar and saturated fats, along with reduced physical activity, are often identified as major drivers of weight gain [1]. Recently, there has been growing awareness of the link between sugar-sweetened beverage (SSB) consumption and weight gain [2]. Extensive meta-analyses suggest that SSB consumption increases the risk of obesity, diabetes, and other health problems [3,4]. While patterns of SSB consumption vary widely among countries, the fastest growth is observed in many low- and middle-income nations [2]. The Scientific Research Report on Dietary Guidelines for Chinese Residents (2021) highlighted that the rapid increase in SSB consumption among Chinese consumers has become a significant public health challenge. Despite the Chinese government intensifying public education campaigns about the health risks of SSBs, there are currently no nationwide regulations on their consumption. However, several pilot policies have been introduced to reduce the public’s intake of added sugar, such as restricting the sale of high-sugar SSBs in primary and secondary schools as well as childcare institutions.

Consumption of SSBs is known to be addictive [5]. Moreover, many consumers addicted to SSBs are well-educated and health conscious. Regulating rational addictive goods like SSBs presents a significant challenge. One of the most common policy measures to curb SSB consumption is the implementation of warning labels. Substantial work has been done to examine the effectiveness of warning labels and the factors influencing their impact [6,7]. Some studies have shown that warning labels significantly reduce SSB consumption [8–10], while others have found no significant effect [7,11]. Despite China’s vast market size, we are not aware of any study investigating Chinese consumers’ reactions to SSB warning labels.

The strength of consumption habits is an important factor influencing consumers’ current consumption [12]. Since individuals vary in their frequency of SSB consumption, their responses to warning labels may also differ. The heterogeneity in consumption frequency may explain the varying effectiveness of SSB warning labels among different people. Previous studies have measured habit strength based on consumption frequency [13–15]. While most research on the impact of habit strength on warning label effectiveness has focused on smoking [16–18], where habit strength significantly influences the effectiveness of cigarette warning labels [19], there has been limited investigation into how habit strength affects responses to SSB warning labels. Considering the importance of habitual behavior in health practices, this study explores how Chinese consumers react to SSB warning labels, with a particular emphasis on the role of habit strength in shaping their responses.

The study contributes to the literature in two aspects: First, it addresses the heterogeneity in consumption frequency and how it leads to different reactions to warning labels. While much research has been done on this topic, little has explored whether individuals with varying habit strengths respond differently to SSB warning labels and how their responses differ. By dividing respondents based on their consumption frequency, we analyze how habit strength influences their reactions to SSB warning labels. Second, we investigate how personal characteristics, including health knowledge and overweight or obesity status, affect the effectiveness of warning labels using sub-samples. The findings from this study are expected to shed light on the roles of habit strength in shaping consumers’ reactions to warning labels. These insights can help policy makers design more targeted policies to regulate the consumption of rational addictive goods, such as SSBs.

## 2. Literature review and hypotheses

### 2.1 The effectiveness of warning labels on SSB purchases

Warning labels, usually placed on the packages of food products, inform consumers of the health risks associated with certain food products through texts or graphics [20,21]. Warning labels are a common public health intervention designed to promote healthier eating habits. Food warning labels generally fall into two categories: nutrient-based warning labels and health-effect warning labels. Nutrient-based labels inform consumers about specific nutrients, while health-effect labels indicate the potential health risks associated with a food product [22].

Warning labels educate consumers about the potential harms of food products, which may alter their attitudes towards health-related behaviors. Being aware of the risk engenders conscious intention to change, which may lead to behavioral change [23]. In addition, warning labels are present at the time of decision making, which may help prime latent health goals and make conscious intention salient [24]. Empirical studies have shown that warning labels changed consumers’ beliefs about the healthiness of food products, which resulted in a change in purchasing intention and behaviors [25,26]. Grummon and Brewer [25] conducted an experiment on adults who knew the adverse health impacts of SSBs. They found that warning labels strengthened the participants’ attitudes towards the adverse health impacts of sugary drinks, weakened their purchase intentions, and ultimately led to a significant reduction in SSB consumption.

Extensive research has been conducted on the effectiveness of warning labels in influencing SSB purchases. Donnelly et al. [8] conducted a field experiment in a cafeteria and found that warning labels made some consumers substitute sugary drinks with mineral water, resulting in a 3.2% decline in SSB consumption. Scully et al. [6] and Billich et al. [27] studied how Australian adults responded to SSB warning labels via an online choice experiment. They found that compared with those who did not see warning labels, participants who noticed warning labels significantly reduced their purchases of SSBs. Hall et al. [28] showed that adding pictorial warning labels to SSBs reduced the likelihood of parents purchasing sugary drinks for their children by 17%, significantly decreasing children’s calorie intake from sugary drinks. Previous studies generally suggest that warning labels on SSBs can evoke negative attitudes and heighten perceptions of risk. Based on this evidence, we propose the following hypothesis:

H1: Implementing health risk warning labels on SSBs decreases consumers’ likelihood of purchasing these products.

### 2.2 The impact of habit on the effectiveness of warning labels

Warning labels promote healthy eating by altering beliefs about the risks associated with certain food products, which may drive a behavioral change. Whether a change in behavior will occur is jointly determined by conscious intention to change and unconscious factors such as habit [12,23,29]. Research indicates that the relationship between intention and behavior is moderated by individual habit strength. Specifically, stronger habits may reduce the impact of intention on behavior [29]. Ji and Wood [12] examined how habits and intentions to change influence behaviors in the contexts of taking bus, buying fast food and watching TV news. They found that conscious intention to change successfully guided behavior only in the absence of strong habits.

Warning labels engender conscious intention to change health-related behaviors. However, behaviors are also affected by unconscious factors such as habits. Gautam et al. [30] discovered that, compared to low-frequency smokers, the presence of warning labels significantly reduced the positive subjective experiences of smoking among high-frequency smokers, but it did not lead to a significant decrease in their smoking duration and amount. Previous studies have primarily focused on distinguishing between strong and weak habits. However, most consumers exhibit moderate habit strength. In this study, we classify habit strength into strong, moderate, and weak categories, and test the following hypothesis:

H2: The effectiveness of warning labels decreases as habit strength increases.

### 2.3 Determinants of the effectiveness of warning labels

Previous studies have suggested that the effectiveness of warning labels is influenced by both the design of the labels and the characteristics of the consumers. Research comparing different warning label designs has shown that factors such as ease of use, interpretability, and salience mattered most to consumers [31]. Miller et al. [22] assessed 27 different warning labels for SSBs and found that labels clearly quantifying sugar content were the most effective in reducing SSB consumption. Grummon et al. [32] indicated that while both health-effect and nutrient-based warning labels can reduce the likelihood of purchasing SSBs, health-effect labels are more intuitive in reflecting the associated risks, making them more effective. Additionally, label characteristics such as color, size, and placement also play a significant role. Studies have shown that colorful [33], large-sized [34], and front of package labels [35] are more likely to capture consumers’ attention and guide them to healthier dietary choices.

The effectiveness of warning labels was also affected by consumers’ personal characteristics. Kim et al. [36] found that warning labels were less effective in improving nutrient intakes of older respondents (aged 60 years and older) compared to younger consumer groups. In contrast, based on a sample of 658 African Americans in North Carolina, Satia et al. [37] showed that warning labels significantly changed the dietary behaviors of older participants, but younger participants were not affected. Yuliati et al. [38] pointed out that gender differences impact responses to self-interested or altruistic messages, with male and female consumers exhibiting varied reactions to warning labels emphasizing different beneficiaries. Aktan [16] concluded that altruism-focused warning labels are more effective for females, whereas self-interest-focused warning labels are more effective for males. Langellier et al. [39] suggested that consumers with higher levels of education tend to be more health-conscious, and are more likely to make healthy dietary choices. In addition, Crockett et al. [40] found that consumers with higher socioeconomic status (SES) pay more attention to warning labels, while those with lower SES are less likely to respond to them.

**Fig 1:**
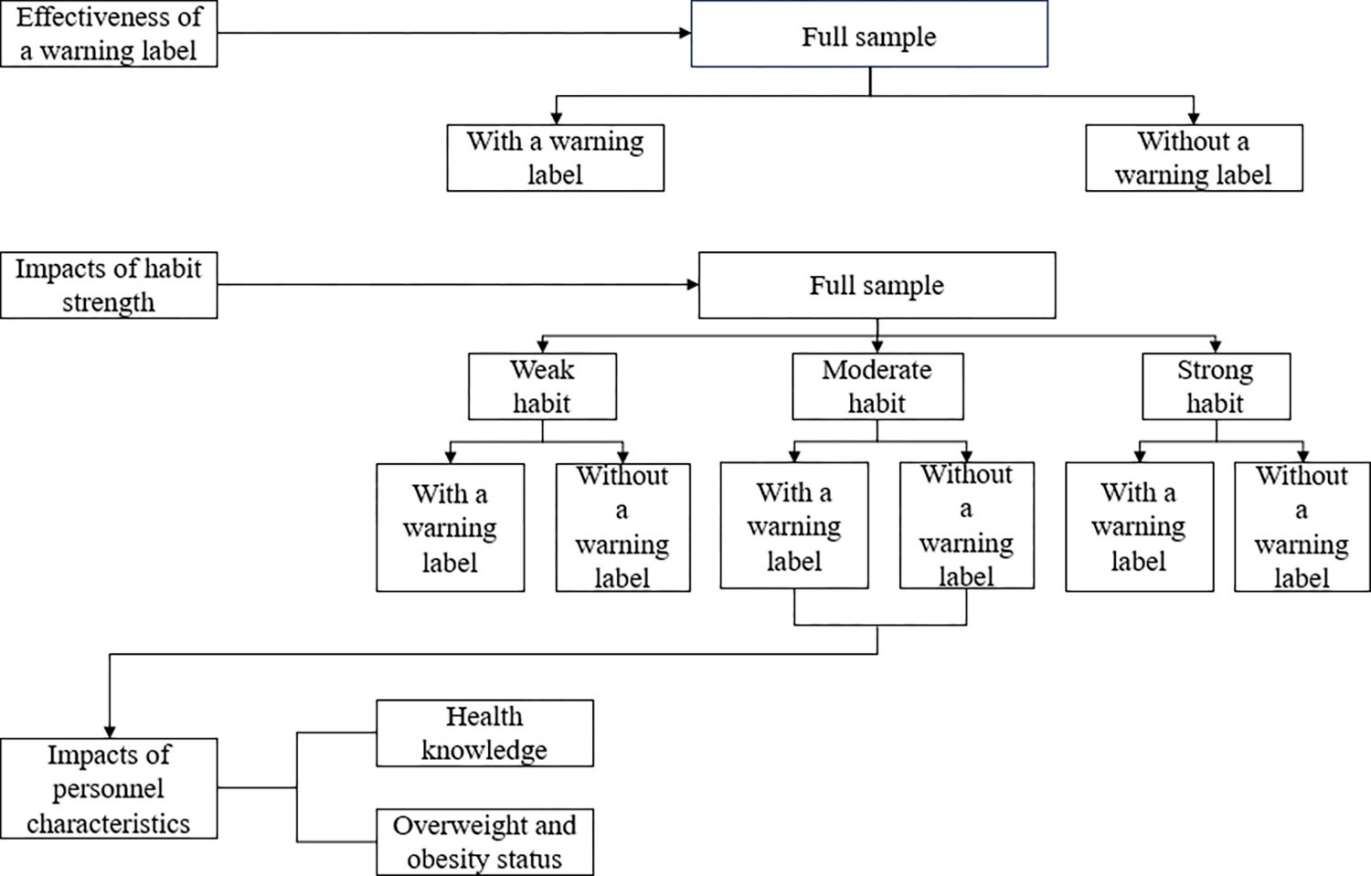
Flow chart of the study.

In the subsequent sections, we utilize choice experiments and random utility models to evaluate the impact of warning labels on SSB consumption. Additionally, we investigate how habit strength and personal characteristics affect the effectiveness of the warning labels. Fig 1 presents a flow chart that illustrates the logical progression of our analyses.

## 3. Methods

### 3.1 Experimental design

We chose carbonated soft drinks as the research subject because of its large market size and popularity among Chinese consumers. Since China has not yet required beverage companies to provide warning labels on SSBs, this study employs a choice experiment to simulate a market scenario in which companies provide warning labels. To identify the warning label effects, we designed two versions of questionnaires. The questions in the two versions are identical except for the choice experiment. In the baseline condition (the control group), the choice experiment is designed to reflect the real market environment in China, with SSBs not carrying a warning label. In the experimental group, the choice experiment simulates a consumption scenario where the government requires companies to place a text warning label on sugary carbonated drinks. Each version of the questionnaire consists of three parts: the first part queries the respondents about their food consumption habits and dietary preferences; the second part presents a choice experiment; the last part contains questions on the respondents’ health behaviors, health status and socioeconomic characteristics.

**Fig 2:**
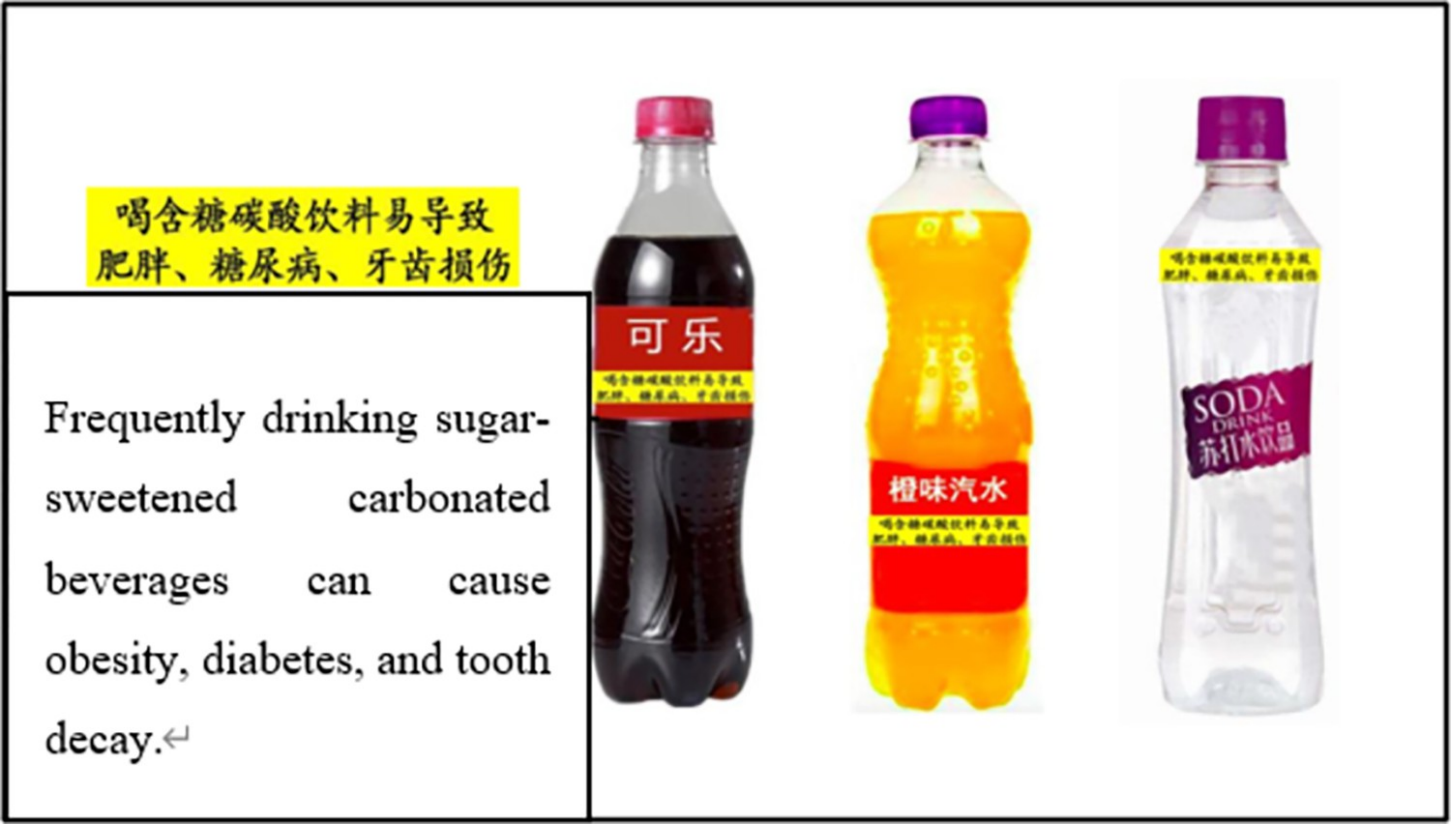
Description of the warning label.

**Fig 3:**
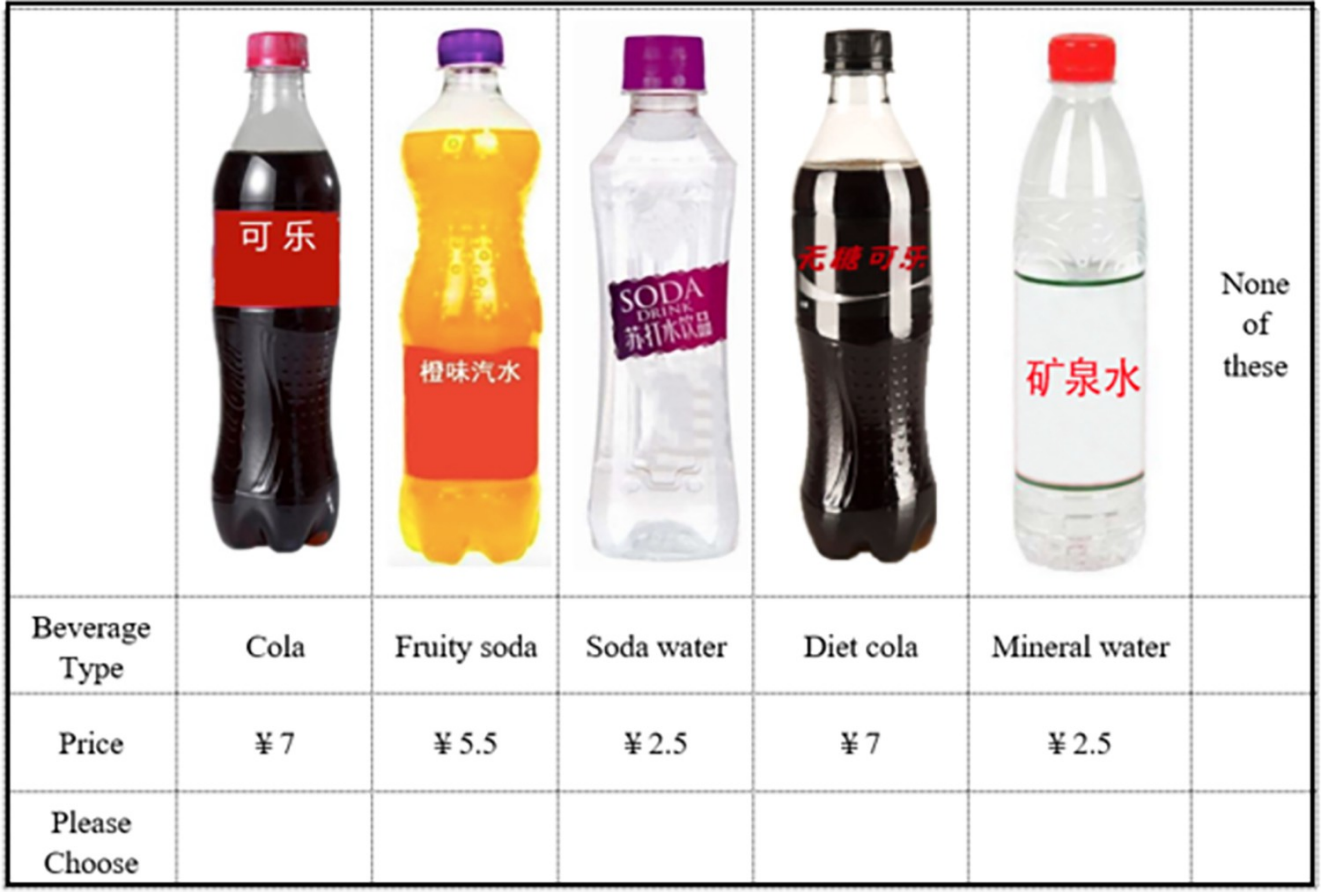
A sample choice question without a warning label.

**Fig 4:**
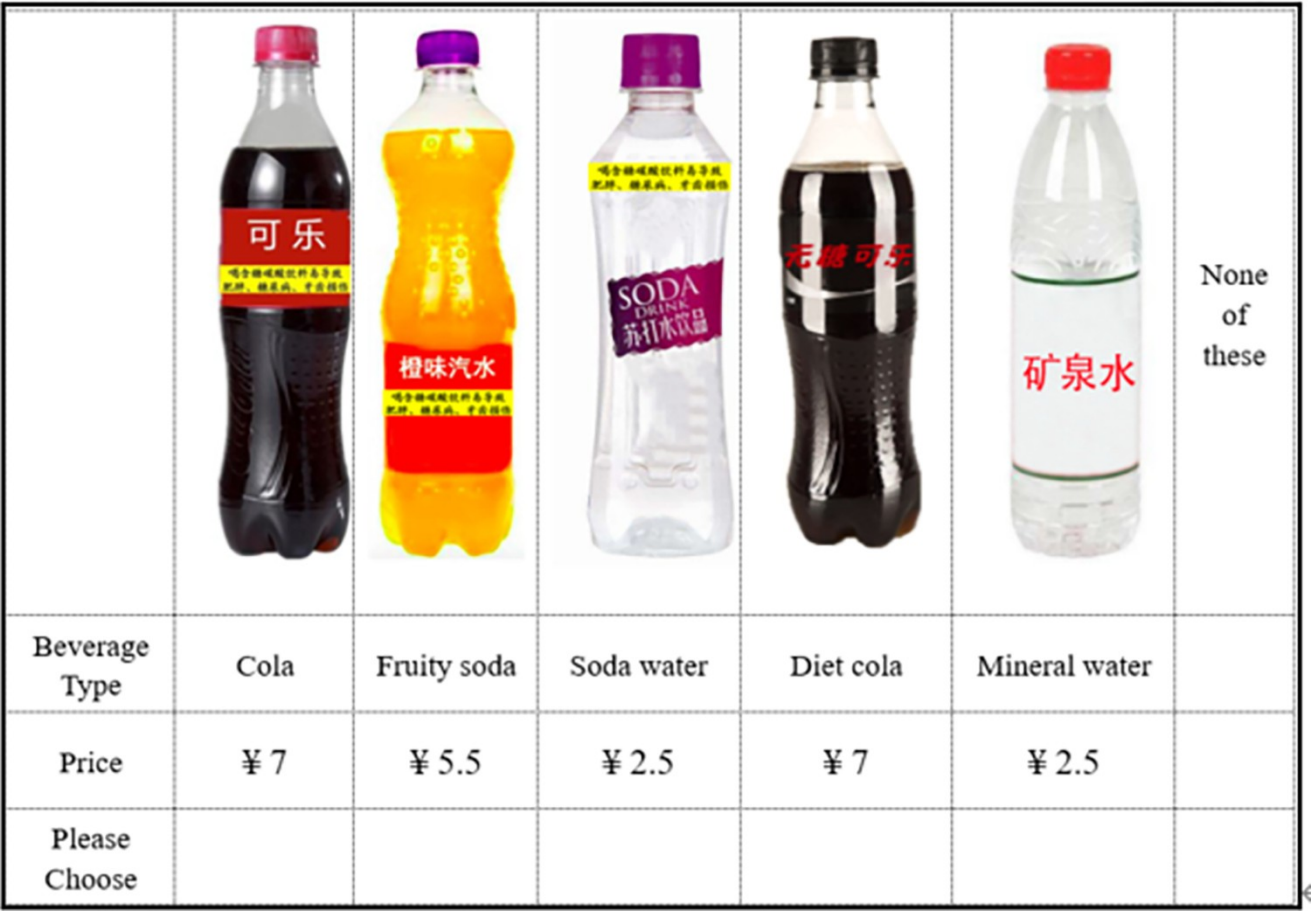
A sample choice question with a warning label.

Choice experiments are a popular tool to study consumer behavior in food retail environments. We define each beverage option by two attributes, beverage type and price, in the choice experiment. The attributes and levels of attributes are presented in Table 1. We use Ngene software to combine different attributes and levels to create six choice sets. Each choice set includes 5 different beverage options and a “no purchase” option. We then divide the six choice sets into three blocks. Each respondent answers two choice questions. As mentioned above, the only difference in the choice experiment between the experimental group and the control group is that a warning label is placed on the front of packages of sugary carbonated beverages in the experimental group. The warning label reads "Frequently drinking sugar-sweetened carbonated beverages can cause obesity, diabetes, and tooth decay" (see Fig 2). A sample choice question in the control group and experimental group are presented in Figs 3 and 4 respectively.

**Table 1:** Product attributes and attribute levels.

Attributes	Attribute levels
Beverage type	Cola, Fruity soda, Soda, Diet cola, Mineral water
Price	RMB 1, RMB 2.5, RMB 4, RMB 5.5, RMB 7, RMB 8.5

We received approval for our study from the Institutional Review Board of the International Food Policy Research Institute (IFPRI) in 2019. We also obtained informed written consent, and the application approval number is DSG-19-1278.

### 3.2 Data collection and descriptive analyses

We conducted online choice experiments in June 2021, administered to an online panel of Chinese urban consumers managed by the survey company Le Survey. The sample was drawn from five major cities across China: Xi’an, Guangzhou, Hangzhou, Chengdu, and Wuhan. Xi’an, Guangzhou, Hangzhou, and Chengdu represent northern, southern, eastern, and western China, respectively, while Wuhan is located in central China. A total of 1200 surveys were completed and deemed valid.

This study targets individuals who consume carbonated beverages. Therefore, we included a screening question at the beginning of the questionnaire to identify participants who had consumed carbonated beverages in the past week. We randomly assigned respondents to the control and experimental group and further randomized the presentation of choice questions within each group. The summary statistics of the sample are given in Table 2. As we can see in Table 2, there are 611 males and 589 females in the sample, accounting for 51.9% and 49.1% of the total sample, respectively. In terms of age distribution, the majority of the sample (77.7%) are in the 25–39 age range, suggesting that consumers of carbonated drinks are mainly people under 40 years old. Liu et al. [41] and Guo et al. [42] also found that consumers under 45 years old are the main force of carbonated beverage consumption in China. As for the education level, the respondents with a bachelor’s degree or above account for 77.8% of the total sample. The reason that the education level of the sample is high is mainly because the cities from which our sample was drawn are homes to many universities. The number of respondents across different annual household income ranges is similar.

**Table 2:** Summary statistics of the sample.

Variables	Sample Size	Percentage (%)
Gender		
Male	611	50.9%
Female	589	49.1%
Age		
24years old or younger	106	8.8%
25–29 years old	317	26.4%
30–34 years old	390	32.5%
35–39 years old	226	18.8%
40 years or above	161	13.4%
Education		
Below post-secondary	46	3.8%
Post-secondary	220	18.3%
Bachelor degree or above	934	77.8%
Annual household income		
RMB 110000 or less	168	14.0%
RMB 110001–130000	148	12.3%
RMB 130001–150000	165	13.8%
RMB 150001–170000	139	11.6%
RMB 170001–190000	123	10.3%
RMB 190001–210000	119	9.9%
RMB 210001–300000	198	16.5%
RMB 300000 or More	140	11.7%

To examine the heterogeneity in the effectiveness of warning labels among individuals with varying habit strengths, we grouped respondents based on their frequency of carbonated beverage consumption. We label those who consume carbonated beverages less than three times per month as “weak habit strength”. The moderate habit strength group consists of respondents who drink carbonated beverages one to six times per week, and respondents who drink carbonated beverages every day are labelled “strong habit strength”. In our sample, 424 respondents exhibit weak habit strength, accounting for 35.33% of the sample; 675 respondents exhibit moderate habit strength accounting for 56.25% of the sample; and 101 respondents exhibit strong habit strength, accounting for 8.42% of the sample.

We are also interested in how individuals with varying habit strengths differ in personal characteristics such as age, income, gender, and health knowledge. We found that approximately 30% of respondents aged 40 or younger exhibit weak habit strength, whereas more than 50% of the respondents older than 40 years exhibit weak habit strength (Table 3). Table 3 also show that compared to consumers with weak and moderate habit, those in the strong habit group have the lowest income levels. There are also differences in habit strengths between male and female consumers. Males tend to consume carbonated beverages more frequently than females. An individual’s health knowledge may also influence the frequency with which they consume carbonated beverages. We measure an individual’s health knowledge based on to what degree the individual agrees/disagrees with the statement “Drinking sugar-sweetened beverages is good for your health” (1 = strongly disagree; 5 = strongly agree). A smaller number indicates better knowledge of the health impacts of drinking SSBs. As we can see in Table 3, respondents who know more about the health impacts of SSBs are less addicted to drinking carbonated beverages. We conducted t-tests to compare the percentages among the weak, moderate and strong habit strength groups. The results indicated significant differences in these percentages across the three groups.

**Table 3:** Socio-demographics of the subsamples with different habit strengths.

Variables	Weak habit	Moderate habit	Strong habit	t1	t2	t3
Below 30 years old	33.25%	57.96%	8.79%	-19.031	53.557	28.957
30–40 years old	32.14%	58.89%	8.97%	-25.054	65.328	33.229
Above 40 years old	52.41%	42.17%	5.42%	4.725	28.084	35.065
Annual household income (in ten thousand CNY)	10.5	10.729	9.347	-2.509	17.402	12.641
Male	31.33%	60.5%	8.17%	-27.110	69.112	33.644
Female	39.33%	49.96%	8.67%	-11.346	56.808	41.883
Health knowledge	2.41	2.54	3.0	-8.929	-41.690	-39.504

Note: t1, t2, and t3 represent the t-values for the comparisons between the following groups: Weak habit vs. moderate habit, moderate habit vs. strong habit, and weak habit vs. strong habit, respectively.

Consumers differ in the frequency with which they drink various types of carbonated beverages. The results in Table 4 show that cola is the most popular among the four listed types of carbonated beverages. Only 3.7% of the sample do not drink cola, which is much lower than the percentage of respondents who do not drink the other three listed types of carbonated beverages. In the meantime, 37.2% of the respondents drink cola 1–6 times per week, and 4.7% of the sample drink cola every day. Soda is the least popular among the four types of carbonated beverages. The percentage of respondents who do not drink soda is the highest (22.3%).

**Table 4:** Consumption frequencies of different types of SSBs.

Variables	No drinking	Less than once per month	1–3 times per month	1–6 times per week	Drinking every day
Cola	3.7%	17.4%	37.1%	37.2%	4.7%
Fruity soda	12.6%	30.3%	40.9%	15.2%	1.1%
Soda	22.3%	31.2%	33.7%	11.9%	1.0%
Diet cola	18.0%	29.6%	33.0%	17.2%	2.3%

## 4. Econometric model

We employ random utility models to analyze the data. The random utility models are based on Lanscaster’s attribute-based utility theory [43], which states that consumers obtain utility from the attributes of a good or service. The random utility model assumes that the utility obtained by individual *n* from product *j* (*U*_*nj*_) is determined by the attributes of the product (*x*_*j*_) and the characteristics of the individual (*z*_*n*_). The utility function is as follows:

$$U_{nj}=V_{nj}+\varepsilon_{nj}$$ (1)

where *V*_*nj*_ is a function of product *j*‘s attributes and individual *n*‘s characteristics, and *ε*_*nj*_ is a random error term. The probability that the decision maker *n* chooses product *j* over product *i* is:

$$P_{nj}=\mathrm{Pr}ob(U_{nj}>U_{ni},\forall i\neq j)=\mathrm{Pr}ob(V_{nj}+\varepsilon_{nj}>V_{ni}+\varepsilon_{ni},\forall i\neq j)$$ (2)

Considering the existence of preference heterogeneity, we employ the random parameters logit (RPL) model to analyze the data. According to the RPL model, the probability of individual *n* choosing product *j* is:

$$P_{nj}=\int \left[\frac{e^{\beta 'x_{j}}}{\sum_{i}e^{\beta 'x_{i}}}\right]f(\beta )d\beta$$ (3)

Where *x* is a vector of product attributes, *β* is a vector of parameters to be estimated, and *f*(*β*) is the probability density function of the parameters. An individual’s marginal willingness to pay (*MWTP*) for a specific attribute can be calculated by:

$$MWTP=-\frac{{\hat{\beta }}_{r}}{{\hat{\beta }}_{p}}$$ (4)

Where ${\hat{\beta }}_{r}$ and ${\hat{\beta }}_{p}$ are the estimated coefficients for the attribute and price, respectively.

In this study, the attributes of carbonated beverages include the type of beverage and price. *V*_*nj*_ takes the form:

$$V_{nj}=\beta_{0}Buynone_{j}+\beta_{1}Coke_{j}+\beta_{2}Fruity_{j}+\beta_{3}Soda_{j}+\beta_{4}Diet_{j}+\beta_{5}\mathrm{Pr}ice_{j}+\beta_{0}Info_{j}$$ (5)

where Cola, Fruity, Soda, and Diet represent four different types of beverages; Info is a dummy variable, taking the value of 1 if the beverage carries a text warning label, otherwise Info equals 0. The definitions of the variables are given in Table 5.

**Table 5:** Definitions of the variables.

Variable	Definition
INFO	Info = 1 if carrying a warning label; 0 otherwise
COLA	Cola = 1 if Cola; 0 otherwise
FRUITY	Fruity = 1 if Fruity soda; 0 otherwise
SODA	Soda = 1 if Soda; 0 otherwise
DIET	Diet = 1 if Diet cola; 0 otherwise
PRICE	Price of a beverage
BUYNONE	Buynone = 1 if choosing “None of these”; 0 otherwise

## 5. Results

### 5.1 Effectiveness of the warning label

We began by assessing whether the text warning label decreases the overall purchases of SSBs across the entire sample. To account for preference heterogeneity, we estimated the RPL model as described in Eq 3. The utility function used in our analysis is defined in Eq 5. The results are shown in Table 6. As can be seen in Table 6, the coefficient on the warning label variable (Info) is negative and significant at the 1% level, suggesting that providing a warning label significantly decreases the purchases of SSBs. The warning label informs consumers of the adverse health impacts of SSBs at the point of purchase, thereby making consumers less likely to choose SSBs. The coefficient of cola is significantly positive at the 1% level, while those of fruity soda, soda and diet cola are not significant, indicating that compared to mineral water, consumers have a strong preference for cola, but they are not significantly more interested in fruity soda, soda and diet cola. The coefficient on price is significantly negative, which means cheaper products are preferred. The coefficient on the no purchase option is significantly negative, which suggests that consuming carbonated beverages increases consumers’ utility relative to not purchasing any beverage.

**Table 6:** The impact of a warning label on carbonated beverage consumption.

Variable	Coefficient	Standard error	95% Confidence interval
Info	-0.304***	0.116	[-0.530, -0.077]
Cola	0.960***	0.110	[0.745, 1.174]
Fruity	0.019	0.138	[-0.251, 0.289]
Soda	0.061	0.138	[-0.209, 0.332]
Diet	0.177	0.112	[-0.042, 0.396]
Price	-0.210***	0.013	[-0.237, -0.184]
Buynone	-6.323***	1.018	[-8.318, -4.329]
N	1200		
Log-likelihood	-3628.797		

Note

***, **, * indicate statistical significance at the 1%, 5% and 10% level, respectively.

### 5.2 Impacts of habit strength on the effectiveness of warning labels

This study focuses on examining the differences in the effectiveness of the warning label among consumer groups with varying levels of habit strength. We expected that consumers with stronger habits would have more difficulty changing their behavior. Since habit strength reflects consumer preference for SSBs, directly including the habit strength variable as an explanatory variable would introduce endogeneity issues. To address this, we grouped consumers based on different levels of habit strength and employed the RPL model described in Eq 3 for each subgroup. This approach allowed us to identify any differences in the impacts of the warning label among these groups. Table 7 presents the effectiveness of the warning label for groups with varying habit strengths.

**Table 7:** The effectiveness of a warning label on carbonated beverage consumption for consumers with different habit strengths.

	Weak habit			Moderate habit			Strong habit		
Variable	Coefficient	MWTP	95% Confidence interval	Coefficient	MWTP	95% Confidence interval	Coefficient	MWTP	95% Confidence interval
Info	0.009 (0.181)	—	[-0.345, 0.364]	-0.530*** (0.159)	—	[-0.843, -0.218]	-0.329 (0.442)	—	[-1.195, 0.537]
Cola	0.063 (0.190)	0.297	[-0.309, 0.435]	1.521*** (0.150)	6.483	[1.227, 1.816]	1.705*** (0.403)	14.643	[0.915, 2.495]
Fruity	-0.636*** (0.239)	-3.000	[-1.103, -0.169]	0.376** (0.191)	1.601	[0.001, 0.750]	1.051** (0.414)	9.025	[0.240, 1.862]
Soda	-0.150 (0.182)	-0.708	[-0.508, 0.207]	0.252 (0.204)	1.075	[-0.148, 0.653]	-0.351 (0.831)	-3.016	[-1.980, 1.277]
Diet	-0.511** (0.204)	-2.408	[-0.910, -0.111]	0.563*** (0.153)	2.40	[0.264, 0.862]	1.217*** (0.335)	10.447	[0.560, 1.873]
Price	-0.212*** (0.023)	—	[-0.256, -0.168]	-0.235*** (0.019)	—	[-0.272, -0.197]	-0.116*** (0.038)	—	[-0.191, -0.042]
Buynone	-6.826*** (1.575)	—	[-9.913, -3.739]	-5.314*** (1.220)	—	[-7.705, -2.922]	-2.268*** (0.523)	—	[-3.294, -1.242]
N	424			675			101		
Log-likelihood	-1324.415			-1972.836			-275.591		

Note

***, **, * indicate statistical significance at the 1%, 5% and 10% level, respectively; standard errors are in parentheses.

The results in Table 7 show that the coefficient of the warning label variable is significantly negative in the moderate-habit group, whereas it is not significant in both the weak-habit and strong-habit groups. This suggests that the warning label only decreases the purchases of SSBs by the consumers with moderate habit. Moreover, the results in Table 7 indicate that consumers varying in habit strengths also differ in their preferences for different types of carbonated beverages. Consumers with weak habit prefer mineral water over the carbonated beverages, whereas consumers in the moderate-habit and strong-habit groups exhibit preference for carbonated beverages except for soda. As we can see in Table 7, the coefficients on soda are not significant in all the three groups. Supporting this, more than 50% of the survey respondents hardly drink soda (see Table 4).

### 5.3 Impact of personal characteristics on warning label effectiveness

The analyses above indicate that warning labels significantly reduce SSB purchases among consumers with moderate habits, but have no effect on the beverage choices of those with weak or strong habits. Consumers with moderate habits comprise 56.25% of the sample, and their SSB choices can be influenced by providing a warning label. Therefore, we further explore the factors affecting the effectiveness of warning labels on these consumers. We focus on an individual’s health knowledge and their overweight or obesity status, as we expect these two factors to influence their response to a warning label.

We measure the respondents’ health knowledge based on their answers to the question “To what degree you agree/disagree with the statement that drinking sugar-sweetened beverages is good for your health?” (1 = strongly disagree, 5 = strongly agree). We create a dummy variable “Wknowledge”. “Wknowledge” takes a value of 1 if the respondents strongly or somewhat agree with the statement; otherwise “Wknowledge” takes a value of 0. We identify whether health knowledge affects the effectiveness of the warning label by including the interaction term between the warning label and “Wknowledge” in Eq 5.

The health awareness and dietary habits of overweight or obese consumers may differ from those who are not overweight or obese [44]. Consequently, these groups may respond differently to the warning label. According to the standards set by China’s National Health Commission, we define overweight and obesity based on an individual’s BMI. Individuals with a BMI greater than 24 are classified as overweight or obese, while those with a BMI of 24 or less are classified as having a normal weight. We create a dummy variable, ’obesity’, which equals 1 if an individual is overweight or obese, and 0 otherwise. To determine if overweight or obese respondents react differently to the warning label compared to those with a normal weight, we include an interaction term between the obesity dummy variable and the warning label variable in Eq 5. The regression results of the RPL model, as specified in Eq 3, are presented in Table 8. The results in Table 8 show that the coefficient on the interaction term between wrong health knowledge and the warning label is significantly positive, indicating that incorrect health knowledge significantly weakens the effect of the warning label. Although the warning label significantly reduces SSB purchases among consumers with moderate habits, the misperceptions about the health impacts of SSB consumption diminish the label’s effectiveness on their purchasing behavior. This suggests that if respondents are unaware of the health risks associated with added sugars or SSBs, warning labels alone are insufficient to alter their misconceptions, thereby reducing the labels’ impact.

**Table 8:** The impact of health knowledge and obesity status on the effectiveness of a warning label.

Variable	Coefficient	Standard error	95% Confidence interval
Info*Wknowledge	0.491**	0.221	[0.058, 0.923]
Info*Obesity	0.709*	0.423	[-0.129, 1.548]
Cola	1.518***	0.150	[1.225, 1.812]
Fruity	0.399**	0.189	[0.029, 0.768]
Soda	0.259	0.203	[-0.139, 0.657]
Diet	0.579***	0.147	[0.291, 0.868]
Price	-0.233***	0.019	[-0.271, -0.196]
Info	-0.845***	0.190	[-1.218, -0.472]
Buynone	-5.263***	1.208	[-7.631, -2.895]
N	675		
Log-likelihood	-1966.910		

Note

***, **, * indicate statistical significance at the 1%, 5% and 10% level, respectively.

Regarding the impact of an individual’s overweight or obesity status, the coefficient on the interaction term between obesity and the warning label is significantly positive. This indicates that it is challenging to alter the beverage choices of overweight or obese consumers solely with a warning label. While the warning label effectively discourages consumers with moderate habit strength from purchasing SSBs, overweight or obese individuals in this group are more likely to continue purchasing SSBs compared to those with a normal weight.

## 6. Discussion

Diet-related chronic diseases impose a significant burden on healthcare systems worldwide, making the promotion of healthy diets a major public health priority in many countries. In June 2021, we collected experimental data through online surveys. Our study finds that adding a textual warning label to SSBs significantly reduced Chinese consumers’ inclination to purchase these beverages, consistent with the findings of Guan et al. [35]. The warning label emphasizes the potential health risks of SSBs, encouraging consumers to prioritize health consideration and opt for healthier beverage alternatives [22,25,26]. Such warning labels hold significant potential to reduce added sugar consumption [45].

Several studies have examined warning label effectiveness from different perspectives. For instance, Hughner and Dumitrescu (2024) demonstrated that beverage companies’ pricing strategies influence the costs consumers incur to adhere to warnings, which in turn moderates the effect of SSB warning labels on consumer behavior [46]. Other research has explored the optimal design and content of warning labels to ensure they effectively capture consumer attention and drive meaningful behavior change [45,47–49]. Building on this literature, our study examines how habit strength affects the effectiveness of warning labels.

We initially expected the effectiveness of warning labels to decrease as consumers’ habit strength increased. However, our findings diverge slightly from this assumption. While previous studies indicated that warning labels are more effective for individuals with weak habits by distinguishing between strong and weak habits [19,30]. In our study, we differentiated between strong, moderate, and weak habit strengths and found that warning labels only led consumers with moderate habit strength to reduce their purchases of SSBs, while having no impact on those with weak or strong habit strength.

A possible explanation is that people with moderate habit strength are more susceptible to external cues and introspective processes. For our study sample, the negative effect of the warning label on purchases outweighs the positive effect of habit, leading consumers with moderate habits to decrease their SSB purchases. For consumers with weak habits, although the warning labels inform them of potential health risks, their infrequent consumption is unlikely to pose a serious health threat, so the labels may not significantly affect their decision-making.

The finding that warning labels have no influence on consumers with strong habit strength aligns with existing literature [30], which may be due to the potentially addictive properties of SSBs [50]. Sugar can produce a strong neurological reward signal that overrides self-control mechanisms [51]. Even though the warning labels inform consumers of the health risks associated with SSB consumption at the point of purchase, the addictive nature of SSBs can lead to entrenched behavior patterns [5]. Additionally, individuals with strong habits may have grown accustomed to disregarding warning information or developed psychological defense mechanisms, leading to resistance towards warning labels [52]. These findings suggest that individual differences exist in the effectiveness of warning labels, underscoring the need for tailored food policies that accommodate varying levels of habit strength among consumers.

An individual’s personal characteristics also significantly influence the effectiveness of warning labels. We found that incorrect health knowledge and overweight or obesity status undermine the effectiveness of warning labels among consumers with moderate habits. Consumers with accurate health knowledge tend to prioritize health impacts when making purchasing decisions, as they understand the potential risks associated with certain foods or products [53]. Consequently, the information conveyed by warning labels is more compelling to them.

Consistent with previous research [54], our study revealed that overweight or obesity diminishes the effectiveness of warning labels. Studies have shown that images and verbal cues associated with high-sugar foods and beverages can trigger stronger preferences and heightened emotional responses, making it more challenging for overweight individuals to resist unhealthy foods [55,56]. Since obesity often results from long-standing unhealthy dietary habits and preferences, relying solely on warning labels is unlikely to effectively reduce the consumption of unhealthy products. Additionally, individuals with obesity are less likely to heed warning labels [54]. These findings underscore the need to consider individual characteristics, such as health knowledge and overweight or obesity status, when designing and implementing warning label policies to promote healthier choices.

Habitual behavior is a crucial aspect of health behaviors. Given that the effectiveness of warning labels varies among consumers with different habit strengths, policymakers should consider individuals’ consumption habits when regulating the SSB market. Consumers with moderate habits represent the majority and their purchasing behaviors are relatively easier to influence. Therefore, policies targeting this group are likely to be more effective. However, our findings indicate that misperceptions about the health impacts of SSBs and overweight or obesity status can weaken the effectiveness of warning labels among consumers with moderate habits. To achieve an effective intervention, policymakers should adopt an integrated approach that combines health education, tax policies, advertising restrictions, and additional measures. For consumers with weak habit strength, increasing education and awareness efforts may be necessary to enhance their health consciousness. The finding that warning labels have no significant impact on the beverage choices of respondents with strong habits suggests that additional interventions are needed to reduce SSB consumption in this group. These interventions could include behavioral therapies, targeted health campaigns, and stricter regulations on SSB marketing and availability.

We acknowledge several limitations in this study. Although choice experiments are widely used in consumer research, the stated choices made in hypothetical scenarios may differ from actual behaviors [45]. Future studies should utilize real market data to test the robustness of our results. Second, there could be endogeneity issues when explaining consumers’ beverage choices using habit strength variables. Due to a lack of suitable instrumental variables, we conducted subsample regressions to mitigate this problem. However, the categorization of habit strengths may be subjective. Additionally, our respondents are urban residents in provincial capital cities in China. Further studies are needed to determine whether the findings can be generalized to a broader population.

## 7. Conclusions

This study employs a choice experiment to simulate a market scenario where a text warning label is presented on SSBs in China. We analyze the effectiveness of the warning label in reducing purchases of SSBs by urban Chinese consumers, with a particular focus on how an individual’s habit strength affects their reaction to the warning label. Our results show that providing a warning label significantly decreases SSB purchases among the sampled consumers. Specifically, the warning label is effective for respondents with moderate habit strength, but it has no impact on those with weak or strong habits. Furthermore, while the warning label can reduce SSB consumption among consumers with moderate habits, its effectiveness is influenced by whether these consumers have correct health knowledge and whether they are overweight or obese. The warning label is more effective for individuals with accurate health knowledge and those who are not overweight or obese. Despite its limitations, our study provides valuable insights into the effectiveness of warning labels and underscores the need for comprehensive strategies that consider individual differences to effectively regulate SSB consumption.

## Supporting information

S1 Dataset Dataset used in this study. (ZIP)
